# Semiconductor laser irradiation improves root canal sealing during routine root canal therapy

**DOI:** 10.1371/journal.pone.0185512

**Published:** 2017-09-28

**Authors:** Dandan Su, Xingxue Hu, Dashan Wang, Ting Cui, Ruyong Yao, Huibin Sun

**Affiliations:** 1 Department of Stomatology, The Affiliated hospital of Qingdao University, Qingdao, China; 2 Division of General Practice and Materials Science, The Ohio State University College of Dentistry, OH, United States of America; 3 Central laboratory, The Affiliated hospital of Qingdao University, Qingdao, China; SRI International, UNITED STATES

## Abstract

**Objective:**

To evaluate the effect of semiconductor laser irradiation on root canal sealing after routine root canal therapy (RCT).

**Methods:**

Sixty freshly extracted single-rooted human teeth were randomly divided into six groups (n = 10). The anatomic crowns were sectioned at the cementoenamel junction and the remaining roots were prepared endodontically with conventional RCT methods. Groups A and B were irradiated with semiconductor laser at 1W for 20 seconds; Groups C and D were ultrasonically rinsed for 60 seconds as positive control groups; Groups E and F without treatment of root canal prior to RCT as negative control groups. Root canal sealing of Groups A, C and E were evaluated by measurements of apical microleakage. The teeth from Groups B, D and F were sectioned, and the micro-structures were examined with scanning electron microscopy (SEM). One way ANOVA and LSD-t test were used for statistical analysis (α = .05).

**Results:**

The apical sealing of both the laser irradiated group and the ultrasonic irrigated group were significantly different from the control group (p<0.5). There was no significant difference between the laser irradiated group and the ultrasonic irrigated group (p>0.5). SEM observation showed that most of the dentinal tubules in the laser irradiation group melted, narrowed or closed, while most of the dentinal tubules in the ultrasonic irrigation group were filled with tooth paste.

**Conclusion:**

The application of semiconductor laser prior to root canal obturation increases the apical sealing of the roots treated.

## Introduction

Pulpitis and periapical periodontitis are the most common oral bacterial infectious diseases in human, with Pain, tooth defect and dysfunction being the major clinical manifestations. Root canal therapy (RCT) is currently the most effective and most commonly provided treatment [1]. The ultimate goal of RCT is to close the root canal system, especially in the last 1/3 of the root tip. RCT prevents bacteria in oral environment from entering and reinfecting the root canal, prevents the tissue fluid from going into the root canal and becoming the residual bacteria culture media, and prevents the occurrence of periodontitis. Therefore, the apical sealing of the root canal is one of the important indicators to a successful RCT [2]. It has been shown that 58.66% of the RCT failure was caused by incomplete root canal closure [3]. Various methods have been used to achieve an optimal root canal closure, such as root canal filling with certain materials after root canal irrigation reverse filling after apicoectomy [4–7].

Because of the three main factors: the complexity of the anatomical structure of the pulp, the biofilm lifestyle and the lavage solution of the colonized bacteria are difficult to penetrate completely through the dentin tubules to the top of the root canal, so the conventional procedure rarely achieves complete roots tube disinfection and closure [8,9]. Microbes can penetrate up to 1mm on dentin tubules, and rinse can only reach about 100μm [9–11]. In addition, due to the ultra-thin diameter of the root and the high surface tension of the root flushing fluid, it is difficult to achieve complete sterilization of the root tip one-third of the root canal.

Previous studies have shown that several laser systems can be used as auxiliary auxiliaries for root canal disinfection [9, 12–15]. Semiconductor lasers are commonly used for dental pulp repair, and their bactericidal properties are mainly related to photothermal effects [12,14, 16–18]. Semiconductor lasers are effective in pulp disinfection due to their affinity for bacterial cells. In addition, laser irradiation can penetrate the dentinal tubules in depth, as it is not absorbed by the hard tissue of the teeth and has reached a 63% reduction in bacterial population at a depth of 750μm [9,12,16–18]. It was showed that after conventional root canal preparation, semiconductor laser irradiation in root canal may help to reduce microleakage and improve root canal closure for a success of root canal therapy. However, few studies on this area have been reported.

So we purpose to evaluate the effects of semiconductor laser irradiation on root canal sealing during routine root canal therapy through this study.

## Materials and methods

### Specimen preparations

Sixty single-rooted teeth with developed periapical areas and similar root length collected due to extractions for orthodontic and periodontal reasons from January 2016 to April 2016. Roots with resorption, fractures or open apices were discarded. Immediately after extraction the teeth were stored in 0.5% chloramines (Beikang, Shantou, China) for 1 month. Informed consents were obtained from the patients. The use of collected teeth has been informed of the patient and oral informed consent also has been obtained from the participants. The experiments were approved by the Institutional Review Board and the Ethical Committee of the affiliated hospital of Qingdao University, and the number of this experiment approved by Institutional Review Board and the Ethical Committee is QYFYKYLL-2016-01-2.

The 60 teeth were randomly divided into 6 groups (n = 10). Groups A and B were the experimental groups, Groups C and D as control group, and Groups E and F as blank group.

### Preparation and obturation of root canal

Each tooth is drilled at the cementum junction through a friction handle with a diamond-coated cylindrical bore under a large amount of water rinse. The canals were accessed, and the length of the teeth was determined by inserting a size #10 stainless steel K-file into the canal until the tip of the file was just visible at the apical foramen. The working length was determined as 1 mm shorter from root apex than the entire length of the teeth. All of the root canals were prepared with Wave-one rotary nickel titanium instruments (X-smart plus, Dentsply, USA) to an apical size of 40, 0.06 taper using 1% sodium hypochlorite (Beikang, Shantou) and17% EDTA (Beikang, Shantou) as the irrigant. The canals were irrigated with 1% sodium hypochlorite (Beikang, Shantou, China) and 17% EDTA (Beikang, Shantou, China) between each file size, and were lubricated with EDTA (Pulpdent, USA). After canal preparation, specimens were rinsed with water, then with 1% NaClO and 17% EDTA (Beikang, Shantou, China), and finally dried with absorbent paper points.

Laser therapeutic instrument (SIROlaser, Sirona, Germany) was used in the study. The Semiconductor laser with 970nm in wavelength and 1Hz–10kHz in frequency delivers energy in pulsed or continuous wave mode, with a maximum output power of 7 W. The 200μr plain ended fibre is suitable for the endodontic application. The continuous wave mode was employed for this laser. The protocol for Semiconductor laser was followed according to the manufacturer’s instructions by a well-trained clinician. The solution was activated by a 970nm wavelength Semiconductor laser (SIROlaser, Sirona, Germany) at 15Hz pulse rate, 20000ms pulse duration and 15000mJ pulse energy, fitted with a newly designed 21-mm-long, 320 microns endodontic conical fibre tip.

After the root canals of Groups A and B were irrigated and dried with paper points, the power of laser therapeutic instrument was set at1W, and the laser optical fiber was placed into the root canal 0.5–1mm from the root tip hole. Each irradiation took 5 seconds with an interval of 5 seconds, and repeated four times [19].

For Groups C and D, as routine procedure of root canal therapy, the 30# ultrasonic irrigation file tip (k30, Satelec, France) was placed into the root till 0.5–1mm from the root apex, pulled back and forth for 1 min rinse.

For Groups E and F, does not make any treatment.

Through the above different processing, sealers (coltene, Switzerland) were mixed according to the manufacturer's directions. The master gutta-percha was coated with sealer and inserted to the full working length, excess gutta-percha was removed with a thermosetting instrument (SybronEndo, USA) after initial set of the sealer. And 3M glassinomer (3M, USA) was placed and sealed on the root canal section.

And then, specimens were stored at 37°C and 100% humidity for 2w to allow the sealer to set.

### Evaluation of radiographs after root canal therapy

Standardized buccolingual and mesiodistal radiographs of the filled roots were taken and evaluated for the root filling situation. The filling materials from the root tip 0.5–2mm from is appropriate; the insufficient or the not compact filling is not filled; the too much filling is overfilling [20]. The overfilling and insufficient filling will be excluded to make sure that the tooth root used in experiment is perfect.

### Apical microleakage detection

Each root of Groups A, C, E was blotted dry and then covered with two coats of nail polish, except for the apical 2 mm. Nail polish was allowed to air-dry for 24h. Experimental and control roots were suspended approximately three-quarters of their length in aqueous 1% basic fuchsia dye. The plates were then placed in an incubator at 37°C for 7 days. At this point, the sample was rinsed under the water for 15 minutes, and the nail polish was removed with a razor. Two opposing longitudinal grooves were made into the dentin on the root surfaces, in order to facilitate the split of the root in half. The depth of the groove cannot be deepened to the root canal by the diamond plate on the buccal side of the sample and the side of the tongue along the long axis of the tooth. The chisel was used to divide the sample longitudinally into two, both of which were used as separate samples. And then observe each part under a stereomicroscope. Each section was then viewed under a stereomicroscope. Linear apical leakage was measured from the apex to the most coronal extent of dye penetration. Evaluate the staining of each part for three times. A reticle calibrated to 0.01mm was used for the measurements.

### Scanning electron microscopy

The tooth root of groups B, D, and F were split longitudinally sectioned with a chisel, the filled gutta-percha in the root canal was removed before examined. One-half of each tooth was selected and prepared for SEM examination.

After the film was polished and polished, the specimens were dried and then placed in a vacuum chamber and sputter-coated with a 300 Å gold layer, The specimens were then observation using a scanning electron microscope (S-4800, Hitachi, Japan). The observation area was selected in the coronal, middle and apical third of the root canal respectively. The root canal wall of the coronal, middle and apical thirds was observed at magnifications of up to ×1200 or ×1500, for the presence/absence of smear layer and visualization of the entrance to dentinal tubules.

### Statistical methods

It should be analyzed with SPSS19.0 statistical software package and the mean value of apical microleakage is shown with the mean ± standard deviation. One way ANOVA and LSD-t test were used for statistical analysis (α = .05).

## Results

### Apical microleakage

To evaluate the effect of laser irradiation on root canal sealing, we collected 60 single-rooted teeth with similar root length collected due to extractions for orthodontic and periodontal reasons. These teeth were randomly divided into 6 groups (n = 10). After different processing each root of Groups A, C, E was subsequently subjected to a dye microleakage assay. The mean values of apical micro leakage are shown with the mean ± standard deviation. Results of microleakage of dye in each group are presented in Table 1.

**Table 1:** Dye microleakage of each group (mm).

Group	Number of samples	Mean	Standard deviation	Pp*
Group A	10	1.70	0.82	p_AE_<0.01
Group C	10	2.02	0.40	p_CE_<0.05
Group E	10	4.56	2.76	

The results show that there was no significant difference in the length of dye microleakage between groups A and C (p> 0.05). By contrast, both roots in group A and the group C showed significant reduction in the length of dye microleakage as opposed to those from group E (p<0.05), although there was no significant difference in apical sealing ability between laser irradiation and ultrasonic washing.

### Scanning electron microscopy reveals improved sealing by laser irradiation

**Fig 1:**
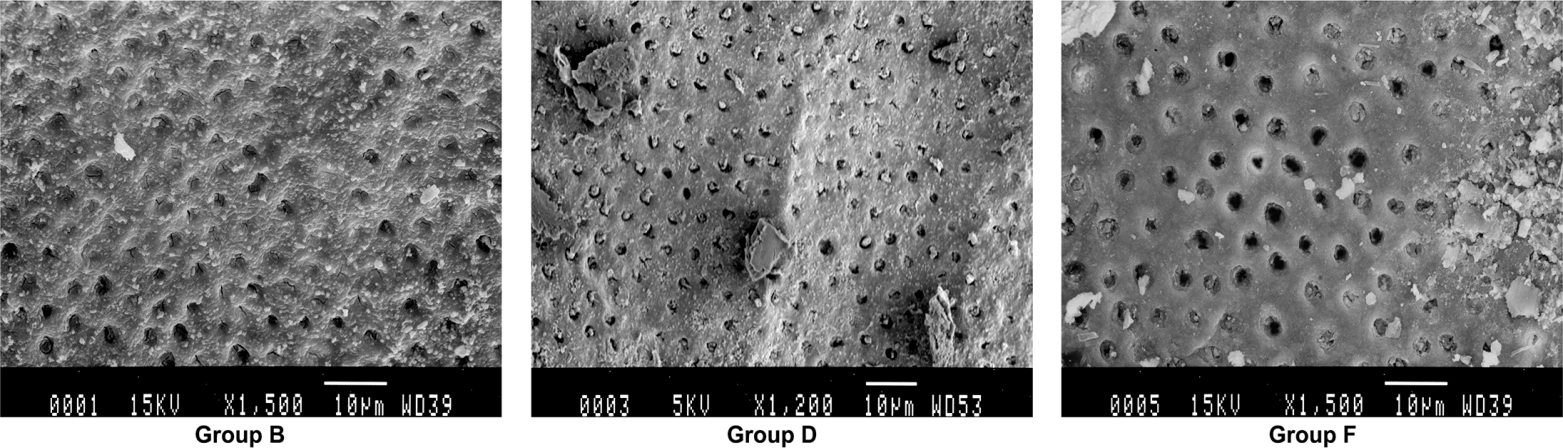
Scanning electron micrographs of the root canals of each group (× 1500).

**Fig 2:**
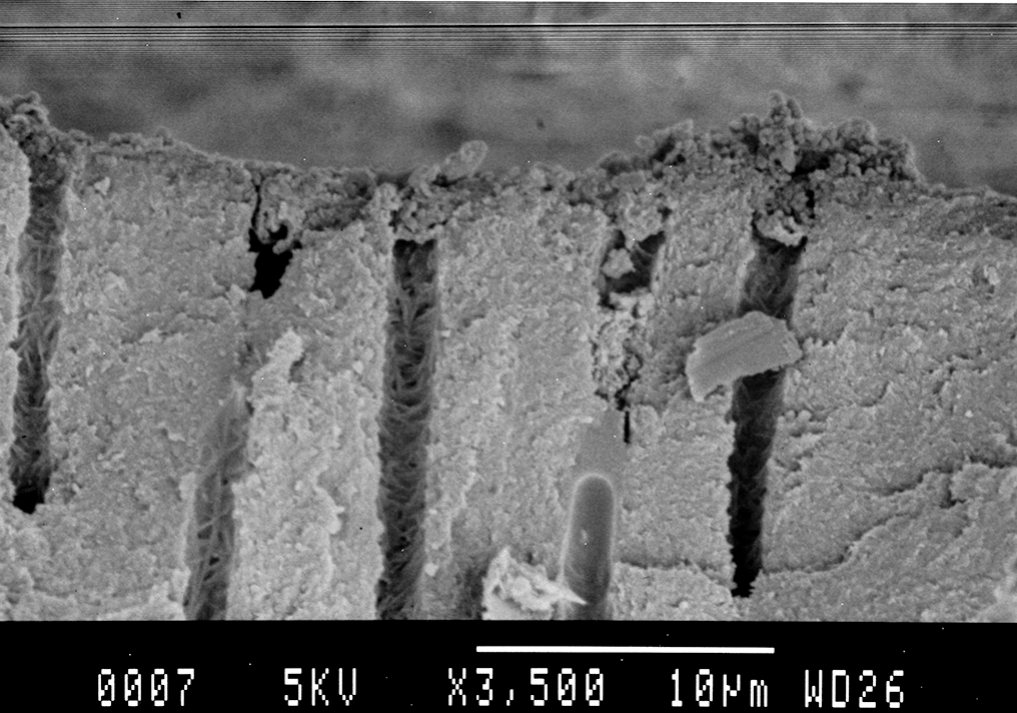
Scanning electron micrographs of the root canals of group B (× 2000) for the laser tube root canal scanning electron microscopy, show that the melting dentin closed the dentinal tubules.

**Fig 3:**
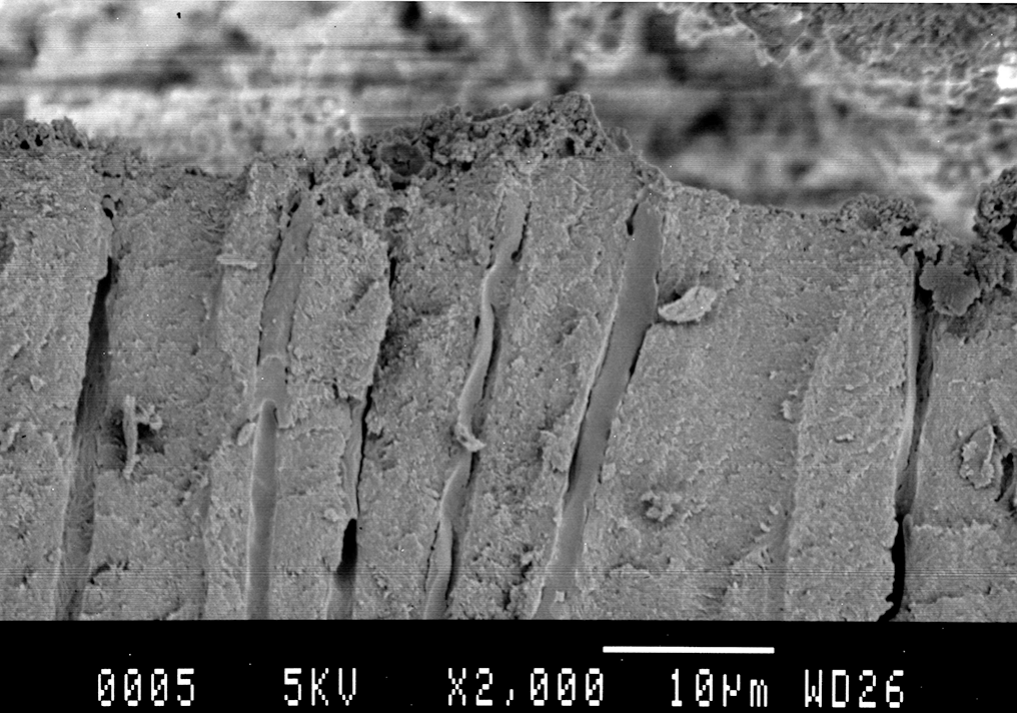
Scanning electron micrographs of the ultrasonic cleaning root canal, show that the gutta-percha into dentinal tubules.

Roots prepared in Group B, D, F were further examined by scanning electron microscope. Shown in Fig 1, most small dentinal tubules in the laser irradiation group B were melted and closed; the number of tubules was reduced. In the ultrasonic cleaning group D, the dentin tubule was clearly visible, and most of the dentin tubules showed gutta-percha or paste. In the control group F, small dentinal tubules were clearly visible, and some dentin tubules showed debris coverage, no or occasionally a small amount of dentin tubules have a paste or gum into. Overall, these data are consistent with that in Table 1 and S1 Table. Next, we performed scanning electron microscopy to evaluate the effect of laser irradiation on the sealing of root canals. Indeed, melting dentin closed the dentinal tubules in this treatment group (Fig 2). By contrast, gutta-percha was found in dentinal tubules of the ultrasonically cleaned root canals (Fig 3).

## Discussion

The prognosis of root canal therapy positively correlates with the degree of root canal filling [21].About 45% of the root canals that are not tightly filled will develop lesions, whereas only 7% of the root canal with tight canal fillings will develop the lesions [22]. As the root canal system is complex and there are many traffic branches and collateral root canal, applying root canal instruments only cannot completely remove infectious substances from the root canal. During root canal preparation, the dentin smear layer will be attached to the root canal wall, which will affect the adhesion of the root-filling material to the root canal wall. It may also lead to apical leakage, causing recurrence of apical infection [23, 24].

At present, the main clinical approach is to use the rinse solution for disinfection and removal of smear layer [25]. EDTA combined with sodium hypochlorite wash is most commonly used. Nui et al. demonstrated that the complete removal of the smear layer requires both organic and inorganic solvents [26]. The combination of sodium hypochlorite and EDTA can remove the organic and inorganic components in the smear layer, so that the dentinal tubules are opened, which can effectively remove the smear layer [27].A previous study [28]found that 17%EDTA combined with 1% sodium hypochlorite rinse effectively removed enterococcus faecalis of the root canal. The bacteria can invade the root canal dentin tubules up to 1mm in depth, while the flushing fluid can only reach the distance of 100μm.Another study [29] found that although the EDTA and sodium hypochlorite alternate flush could effectively remove the neck 1/3 and middle 1/3 of the smear layer wall, it performed poorly in removing the root tip 1/3 smear layer. Because of the narrow lumen of the apical third of the root, it is difficult for the flushing fluid to reach, and to maintain an effective concentration. In addition, the flushing effect of sodium hypochlorite was overestimated in the trial, because the clinical form of the root canal was much more complex than the single root canal used in the previous study [9].Therefore, conventional root canal preparation combined with irrigation fluid will achieve the perfect root canal filling effect [30].

Ultrasonic cleaning is developed in recent years, and it is a fast and effective physical cleaning methods. In the solution, the ultrasonic file produces the instantaneous cavity effect, the sound flow effect, the synergistic effect in the root canal, and the effect of heating and agitation and hence achieves the removal of the root canal smear and dentin debris. Li Yuliang et. al. [31] has shown that ultrasonic washing effectively improved the success rate of root canal therapy. However, the number of dentinal tubules in the apical region was small and the opening was small, root canal diameter gradually reduced from the root canal orifice to apical area. The amount of washing liquid reaching the apical region was small and the reflux was poor. The smear layer removal effect of ultrasonic cleaning on the neck 1/3 and middle 1/3 is better, but it is less effective on the aplical third. Therefore, ultrasonic cleaning cannot completely remove the root canal smear layer [32,33].

At present lasers have been widely used in oral soft tissue surgery, mucosal disease, periodontitis and dental pulp diseases and other fields, A number of studies have indicated that after the root canal preparation, the semiconductor laser irradiation in the root canal may reach the area of the root canals which the conventional disinfection fail to enter [34]. Semiconductor laser irradiation may carry out multiple functions, including disinfection and sterilization, removal of the smear layer, and melting and sealing of the dentin tubule to improve the sealing performance of the root canal [35,36].

The present study was designed to evaluate the effect of semiconductor laser irradiation on root canal obturation after routine root canal therapy. Many methods have been used to evaluate the sealing ability of filling materials, but the most common one involves dyes and radioactive isotopes. Among dyes, 1% basic fuchsia dye is widely used for convenience and can provide a high degree of penetrability due to its small molecular weight [37]. Many researchers have used dyestuffs to detect microleakage of root-filling materials [38, 39].

The apical sealing after root canal filling was compared between the three groups, namely the laser irradiation root canal after root canal preparation group, ultrasonic cleaning group and untreated group. Overall both laser irradiation and ultrasonic washing significantly improved the sealing performance compared with the blank group (p<0.05), and that laser irradiation combined with conventional root canal preparation is superior in improving the performance of root canal sealing (p<0.01).

The results of scanning electron microscopy in this study showed that most small dentinal tubules in the laser irradiation group were melted and closed, the number of tubules was also reduced, the edge was blunt and thickened, and some molten crystals were embedded on the surface. Some dentin tubules were sealed by molten dentin (Fig 1B, Fig 2).In the ultrasonic cleaning group, the dentin tubule was clearly visible, and most of the dentin tubules showed gutta-percha or paste (Fig 1D, Fig 3).In the blank group, small dentinal tubules were clearly visible, and some dentin tubules showed debris coverage (Fig 1F). Our results are consistent with the results of semiconductor laser-assisted root canal therapy [36].In the study by Ravalli et al. [40], after semiconductor laser irradiation the root canal showed that the surface was smooth and clean, and there are a small amount of dentin tubules exposed and large area of dentin tubule melting. Marc et al. used a laser to irradiate the hydroxyapatite powder, which had been previously placed in the apical foramina [41]. The hydroxyapatite powder melts and the apical foramen can be closed. Our results demonstrated that the semiconductor laser irradiation of the root canal effectively removed the smear layer. The semiconductor laser irradiation also causes the occlusion of the dentinal tubules and prevents the residual material in the root canal from entering the periapical space through the apical or dentinal tubules which may reduce the occurrence of periapical lesions. These changes will likely prevent re-infection caused by bacteria, which could have entered the root canal through the dentinal tubules, and improve the success rate of root canal treatment. What’s more laser irradiation and ultrasonic swing can effectively improve the closure of the root canal, so there is no difference in microleakage detection. But the principle of the apical sealing is different, so there are differences in the SEM image.

Our experiments also show that the root canal filling after laser irradiation failed to achieve complete closure of the root canal system. A possible cause could be the complexity of the root canal system. In the present study the laser cannot reach all parts of the root canal system, especially the root canal and curved root canal root tips, which greatly affect the bactericidal effect.

Thermal injury of periodontal tissue is a major concern of laser therapy in root canal therapy. Laser irradiation in the root canal dentin may increase the temperature of the external and adjacent tissue of the root canal. This temperature change may induce root and periodontal ligament fibrous absorption, alveolar bone necrosis and pain. Alfredo et al [42] used a pulsed mode of 1.5 w or 3.0 w for 20 s which made the temperature increase of root canal wall less than 10, and it had no significant effect on periodontal tissue. Therefore, as long as the appropriate laser settings are selected, the effect of the heat generated by the laser on the periodontal tissues is very small, and the heat it produces may also be relieved by the root canal irrigant.

Application of semiconductor laser in root canal therapy effectively removes the smear layer within the root canal. It allows the root canal filling material and the root canal tightly integrated. It also improves root canal closure through melting and remineralization of hard tissue and closed dentinal tubules. However, discrepancies are also noticed among different studies because of the inconsistent laser parameters and experimental conditions used in each study. The application of semiconductor laser in clinics is just being explored. Therefore, more standardized experiments are needed to optimize the application of laser in clinic. Nevertheless, results from our study now reveal the great the promise of clinical application of laser during RCT.

Results from our study demonstrated the application of semiconductor laser prior to root canal obturation increases the apical sealing of the roots treated.

## Supporting information

S1 Table Dye penetration length of each sample (mm). Relevant data underlying the findings described in manuscript.
(DOCX)

## References

[1] Mingwen Fan, et al Endodontics. People's Health Publishing House, 2012:260.

[2] Wu Xiaozhen, Min Yu, Gong Lin. Compare study of apical sealing in cold lateral condensation using gutta-percha with different tapered angles. Journal of Clinical Stomatology, 2014, 30(5):294–296.

[3] Peters OA, Schönenberger K, Laib A. Effects of four Ni-Tiprepa⁃ration techniques on root canal geometry assessed by micro computed tomography [J]. Int Endod J, 2001, 34(3):221–230. 12193268 10.1046/j.1365-2591.2001.00373.x

[4] Bawazir O A, Salama F S. Apical microleakage of primary teeth root canal filling materials [J]. Journal of Dentistry for Children, 2007, 74(1):46–51. 18430355

[5] Eldeniz A U, Mustafa K, Ørstavik D, et al Cytotoxicity of new resin-, calcium hydroxide- and silicone-based root canal sealers on fibroblasts derived from human gingiva and L929 cell lines.[J]. International Endodontic Journal, 2007, 40(5):329–37. doi: 10.1111/j.1365-2591.2007.01211.x 17309743 10.1111/j.1365-2591.2007.01211.x

[6] Kuah H G, Lui J N, Tseng P S, et al The effect of EDTA with and without ultrasonics on removal of the smear layer [J]. Journal of Endodontics, 2009, 35(3):393–396. doi: 10.1016/j.joen.2008.12.007 19249602 10.1016/j.joen.2008.12.007

[7] Townsend C, Maki J. An in vitro comparison of new irrigation and agitation techniques to ultrasonic agitation in removing bacteria from a simulated root canal. [J]. J Endod, 2009, 35(7):1040 doi: 10.1016/j.joen.2009.04.007 19567330 10.1016/j.joen.2009.04.007

[8] Nair PNR, Henry S, Cano V, Vera J. Microbial status of apical root canal system of human mandibular first molars with primary apical periodontitis after ‘one-visit’ endodontic treatment. Oral Surg Oral Med Oral Pathol Oral RadiolEndod 2005; 99: 231–52. 10.1016/j.tripleo.2004.10.005 15660098

[9] Romeo U, Palaia G, Nardo A, et al Effectiveness of KTP laser versus 980 nm diode laser to kill Enterococcus faecalis in biofilms developed in experimentally infected root canals. Aust Endod J, 2015, 41(1):17–23. doi: 10.1111/aej.12057 24588799 10.1111/aej.12057

[10] Berutti E, Marini R, Angeretti A. Penetration ability of different irrigants into dentinal tubules. J Endod 1997; 23: 725–7. doi: 10.1016/S0099-2399(97)80342-1 9487845 10.1016/S0099-2399(97)80342-1

[11] Peters LB, Wesselink PR, Buijs JF, van Winkelhoff AJ. Viable bacteria in root dentinal tubules of teeth with apical periodontitis. J Endod 2001; 27 (2): 76–81. doi: 10.1097/00004770-200102000-00002 11491642 10.1097/00004770-200102000-00002

[12] Schoop U, Kluger W, Moritz A, Nedjelik N, Georgopoulos A, Sperr W. Bactericidal effect of different laser systems in the deep layers of dentin. Lasers Surg Med 2004; 35: 111–6. doi: 10.1002/lsm.20026 15334613 10.1002/lsm.20026

[13] Moritz A, Jakolitsch S, Goharkhay K et al Morphologic changes correlating to different sensitivities of Escherichia coli and Enterococcus faecalis to Nd:YAG laser irradiation through dentin. Lasers Surg Med 2000; 26 (3): 250–61. 10738287 10.1002/(sici)1096-9101(2000)26:3<250::aid-lsm2>3.0.co;2-h

[14] Pirnat S, Lukac M, Ihan A. Study of the direct bactericidal effect of Nd:YAG and diode laser parameters used in endodontics on pigmented and nonpigmented bacteria. Lasers Med Sci 2011; 26: 755–61. doi: 10.1007/s10103-010-0808-7 20582609 10.1007/s10103-010-0808-7

[15] Upadya MH, Kishen A. Influence of bacterial growth modes on the susceptibility to light-activated disinfection. Int Endod J 2010; 43: 978–87. doi: 10.1111/j.1365-2591.2010.01717.x 20722757 10.1111/j.1365-2591.2010.01717.x

[16] Gutknecht N, Franzen R, Schippers M, Lampert F. Bactericidal effect of a 980-nm diode laser in the root canalb wall dentin of bovine teeth. J Clin Laser Med Surg 2004;22 (1): 9–13. doi: 10.1089/104454704773660912 15117481 10.1089/104454704773660912

[17] De Souza EB, Cai S, Simionato MR, Lage-Marques JL. High-power diode laser in the disinfection in depth of the root canal dentin. Oral Surg Oral Med Oral Pathol Oral Radiol Endod 2008; 106 (1): e68–72. doi: 10.1016/j.tripleo.2008.02.032 18585615 10.1016/j.tripleo.2008.02.032

[18] Preethee T, Kandaswamy D, Arathi G, Hannah R. Bactericidal effect of the 908 nm diode laser on Enterococcus faecalis in infected root canals. J Conserv Dent 2012; 15: 46–50. doi: 10.4103/0972-0707.92606 22368335 10.4103/0972-0707.92606 PMC3284013

[19] Meire M A, De Prijck K, Coenye T, et al Effectiveness of different laser systems to kill Enterococcus faecalis in aqueous suspension and in an infected tooth model[J]. International Endodontic Journal, 2009, 42(4):351–9. doi: 10.1111/j.1365-2591.2008.01532.x 19220514 10.1111/j.1365-2591.2008.01532.x

[20] Mingwen Fan, et al Endodontics. People's Health Publishing House, 2012:317.

[21] Sjogren U, Hagglund G, Sundqvist G, et al Factors affecting thelong-term results of endodontic treatment [J]. J Endod. 1990, 16(10):498–504. doi: 10.1016/S0099-2399(07)80180-4 2084204 10.1016/S0099-2399(07)80180-4

[22] Fleming CH, Litaker MS, Alley LW, et al Comparison of classic endodontic techniques versus contemporary techniques on endodontic treatment success[J].J Endod,2010,36(3):414–418. doi: 10.1016/j.joen.2009.11.013 20171354 10.1016/j.joen.2009.11.013

[23] Violich DR, Chandler NP. The smear layer in endodontics—a review. Int Endod J, 2010,43(1):2–15. doi: 10.1111/j.1365-2591.2009.01627.x 20002799 10.1111/j.1365-2591.2009.01627.x

[24] Stabholz A, Sahar-Helfl S, Moshonov J. The use of lasers for cleaning and disinfecting of the root canal system. Alpha Omegan,2008,101(4):195–201. 19166084 10.1016/j.aodf.2008.07.029

[25] Lottanti S, Gautschi H, Sener B, Zehnder M. Effects of ethylenediaminetetraacet-ic, etidronic and peraceticacid irrigation on human root dentine and thesemear layer. Int Endod J. 2009 4;42(4):335–43. doi: 10.1111/j.1365-2591.2008.01514.x 19220516 10.1111/j.1365-2591.2008.01514.x

[26] Niu W, Yoshioka T, Kobayashi C, et al A scanning electron microscopic study of dentinal erosion by final irrigation with EDTA and NaOCl solutions. Int Endod J,2002,35(11):934–939. 12453023 10.1046/j.1365-2591.2002.00594.x

[27] Chang Liu, Hong Liu, Chunying Yang. Impact of six different irrigations on ultra-structure of post space [J].Journal of Practical Stomatology, 2010, 26(5): 650–654.

[28] Peters LB, Wesselink PR, Buijs JF, et al Viable bacteria in root dentinal tubules of teeth with apical periodontitis. J Endod 2001, 27(2):76–81. doi: 10.1097/00004770-200102000-00002 11491642 10.1097/00004770-200102000-00002

[29] Xiao Zhang, Jie An, Jingyuan Liu. Influence of different chemical irrigants on the dentin surface morphology of post space [J]. Journal of Practical Stomatology, 2013, 29(1):101–104.

[30] Lee SJ, Wu MK, Wesslink PR, et al The effectiveness of syring irrigation and ultrasonicsto remove debris from simulated irregularities within prepared root canal walls. Int Endod J. 2004 10;37(10):672–8. doi: 10.1111/j.1365-2591.2004.00848.x 15347291 10.1111/j.1365-2591.2004.00848.x

[31] Yuliang Li. Application of P5 ultrasonic therapeutic apparatus in root canal therapy of posterior [J].Journal of Practical Medical Techniques, 2011, 18(11):1153–1154.

[32] Hui Zhao, Guangshui Jiang, Jun Cai, et al To compare the effect of ultrasonic treatment with different stalls and different concentrations of sodium hypochlorite root canal [J]. Shandong Medical Journal, 2015, 55(13):86–87.

[33] Schoop U, Kluger W, Moritz A. Bactericidal effect of different laser systems in the deep layers of dentin. Lasers Surg Med. 2004; 35(2):111–6. doi: 10.1002/lsm.20026 15334613 10.1002/lsm.20026

[34] Siqueira JF Jr, Rôças IN, Favieri A, et al Chemomechanical reduction of the bacterial population in the root canal after instrumentation and irrigation with 1%, 2%, and 5.25% sodium hypochlorite. J Endod. 2000 6;26(6):331–4. doi: 10.1097/00004770-200006000-00006 11199749 10.1097/00004770-200006000-00006

[35] Schoop U, Kluger W, Moritz A, et al Bactericidal effect of different laser systems in the deep layers of dentin. Lasers SurgMed, 2004, 35(2): 111–116 10.1002/lsm.20026 15334613

[36] Wanghong Li, Dongxiao Yang. Experimental study on root canal therapy by diode laser[J]. Zhejiang Medical Jouranl, 2001, 23(12):726–727.

[37] Becker SA, Van Fraunhofer JA. The comparative leakage behavior of reverse filling materials. J Endo 1989;15:246–8. 10.1016/S0099-2399(89)80217-1 2592878

[38] Ozata F, Onal B, Erdilek N, et al A comparative study of apical leakage of Apexit, Ketac-Endo, and Diaket root canal sealers[J]. 1999, 25(9):603–604. doi: 10.1016/S0099-2399(99)80317-3 10687537 10.1016/S0099-2399(99)80317-3

[39] Rohde TR, Bramwell JD, et al An in vitro evaluation of microleakage of a new root canal sealer. J Endod. 1996 7;22(7):365–8. doi: 10.1016/S0099-2399(96)80220-2 8935063 10.1016/S0099-2399(96)80220-2

[40] Radaelli C M, Zezell D M, Cai S, et al Effect of a high power diode laser irradiation in root canals contaminated with Enterococcus faecalis. “In vitro” study[J]. International Congress, 2003, 1248(1248):273–276.

[41] Mor C, Stabholz A, Neev J, et al Efficacy of XeCL-308 excimer laser in fusing hydroxy apatite to seal the root apex. Endod Dent Traumatol, 1995, 11(4):169–71. 7588339 10.1111/j.1600-9657.1995.tb00481.x

[42] Alfedo E, Marchesan MA, Sousa—Neto MD, et a1 Temperature variation at the external root surface during 980–nm diode laser irradiation in the root canal[J]. J Dent, 2008, 36(7):529–534. doi: 10.1016/j.jdent.2008.03.009 18462858 10.1016/j.jdent.2008.03.009

