# Supplementary material for: Semiconductor laser irradiation improves root canal sealing during routine root canal therapy
Source: PLoS One. 2017 Sep 28;12(9):e0185512. doi: 10.1371/journal.pone.0185512 (PMC5619785; doi:10.1371/journal.pone.0185512)
Supplement: S1 Table — Relevant data underlying the findings described in manuscript. (DOCX) [file pone.0185512.s001.docx]

supporting information

S1 Table. Relevant data underlying the findings described in manuscript

|  |  |  |  | Tooth | number |  |  |  |  |  |
| --- | --- | --- | --- | --- | --- | --- | --- | --- | --- | --- |
|  | NO.1 | NO.2 | NO.3 | NO.4 | NO.5 | NO.6 | NO.7 | NO.8 | NO.9 | NO.10 |
| Group A  Group C  GroupE | 2.12  2.06  2.28 | 2.20  2.47  5.56 | 0.84  2.24  1.94 | 1.03  3.00  4.88 | 1.16  1.32  11.00 | 3.69  2.06  1.76 | 1.79  1.62  1.50 | 0.83  2.29  1.95 | 0.53  1.94  4.14 | 2.79  1.24  1.95 |
